# Checklist for transferability of multidisciplinary evaluations of telemedicine

**Published:** 2012-06-15

**Authors:** Anne-Kirstine Dyrvig, Kristian Kidholm

**Affiliations:** Odense University Hospital, Denmark; Odense University Hospital, Denmark

**Keywords:** transferability, evaluation, health technology assesment, HTA, checklist

## Abstract

**Introduction:**

Emerging opportunities for the use of new technologies leads to an increasing need for timely and qualified input for decision-making in hospitals and the health care sector. Health Technology Assessment (HTA) is a useful tool for decision-makers to assess the effects of introducing a new technology. Full HTA evaluations are, however, resource and time consuming due to the methodological demands of multiple scientific fields. Thus, it is necessary to be able to transfer results between settings, with some kind of adjustment to local prerequisites, in order to limit the number of assessments needed and to accommodate the need for timely information on the effects of telemedicine applications.

**Aims and objectives:**

The overall aim of this project is to develop a checklist for the transferability of results from HTAs of telemedicine. Based on comparisons of the empirical data from three RCTs on the effects of telemedicine for COPD patients, a study will be carried out indicating which variables on organisational and other aspects to consider in order to extrapolate results to other settings.

**Methods:**

The common outcome measures from the three RCTs will be compared in two different ways in order to explore the differences between the local settings:

- Individual patient data (IPD) from the three trials will be combined in an IPD meta analysis, and the explanation for variance will be explored through multiple regressions with the outcome as dependent variable and possible effect modifiers as independent variables.
- Quantitative summary measures, i.e. odds ratios will be combined in meta analysis and in cases of heterogeneity, a random effects model will be used and the heterogeneity will be explored through meta regressions. Also, possible intracluster correlations will be explored through multilevel regression analyses.

**Results:**

The fact that the study includes data from three settings permits quantitative analyses of the impact of organisational aspects on treatment effects. An operationalization of organisational aspects identifies independent variables for the regression analyses i.e. hospital size, staffs’ attitude and knowledge on IT, level of education among staff, communication among staff etc. The dependent variables are the primary and secondary outcomes of the RCTs, i.e. number of readmissions, patients’ quality of life, use of resources etc. Independent variables explaining the variance in between-country-results or between-strata-results with predefined strata (e.g. based on age, income, use of telemedicine service) being defined through the organisational analysis. These analyses will result in identification of how much variance in the common results is explained by which independent variable. This will provide a picture of which variables are necessary and in what form they should be presented (dichotomous, numerical or categorical), if they should be extrapolated to another setting.

**Conclusions:**

This study will result in a checklist for transferability of results of telemedicine evaluations that can serve as a guide for what to present in articles for others to be able to implement similar interventions and know what to expect in terms of results without reproducing a costly HTA.

